# Book Reviews

**Published:** 1873-05-15

**Authors:** 


### Book Reviews.

Civil Malpractice—a Report presented to the Military Tract Medical Society, Jan. 14th, 1873. By M. A. McClelland, M.D. Chicago: W. B. Keen, Cook & Co. Price $2.00.

This report, reprinted from late numbers of the Chicago *Medical Journal*, forms a small volume of 74 pp., substantially bound in cloth. Itconstitutes a brief, concise treatise upon this important and too much neglected subject.

The definitions and decisions, derived from legal sources, are transcribed in full. The quotations from medical works, however, have been abridged as much as possible, the works from which they are derived being more generally accessible to the people.

A Handbook of Medical Electricity. By Herbert Tibbits, M.D., L.R.C.P., with 64 illustrations. Philadelphia: Lindsay & Blakiston. $2.25.

This is a volume of 100 pages, in neat cloth binding. The author claims no especial originality for his work, but only an earnest endeavor to sift the wheat of our existing knowledge from the chaff, and to make his reader as much at home with his electrical as with his other medical instruments; and further, to lead him to estimate electricity at its fair and proved value in therapeutics, as an agent not to be indiscriminately advocated as a panacea, nor, on the other hand, neglected by the inexperienced, but, in appropriate cases, to be regarded as one of the most powerful and serviceable weapons with which we can combat disease.

